# Renal Infarction in a Newly Diagnosed Atrial Fibrillation Patient

**DOI:** 10.7759/cureus.73457

**Published:** 2024-11-11

**Authors:** Hogir Aldawoody, Amir Thakur

**Affiliations:** 1 Emergency Medicine, St. Mary's Hospital, Isle of Wight NHS Trust, Isle of Wight, GBR

**Keywords:** atrial fibrillation, contrast enhanced ct abdomen, flank pain, renal infarction, thromboembolism

## Abstract

Renal infarction is a rare but potentially serious condition, often misdiagnosed due to its non-specific presentation, which mimics conditions such as nephrolithiasis and pyelonephritis. Discussed in this case report is a 68-year-old woman who presented to an emergency department with two weeks of worsening left flank pain. She was found to have a new diagnosis of atrial fibrillation (AF) on electrocardiogram (ECG) and CT findings revealing renal infarction. The patient was effectively managed with anticoagulation, pain relief, and outpatient vascular surgery follow-up. Early identification was crucial to mitigating complications such as renovascular hypertension and chronic kidney disease. This case underscores the importance of maintaining clinical suspicion for renal infarction in patients presenting with unexplained flank pain in the setting of new-onset AF. Our report highlights the value of a thorough history, physical examination, and timely imaging. These factors ensure both optimal diagnostic accuracy and improved patient-centered outcomes with this rare pathology.

## Introduction

Renal infarction occurs when the blood supply to the kidney is suddenly interrupted, leading to ischemia and tissue necrosis. It is a rare but serious condition that often presents with non-specific symptoms, making diagnosis challenging. The most common causes include thromboembolism, often from cardiac sources such as atrial fibrillation or valvular heart disease, and in situ thrombosis due to conditions like renal artery stenosis or atherosclerosis. Due to its unusual presentations, the diagnosis is usually missed or delayed, which may lead to complications such as chronic kidney disease and end-stage renal failure [1]. In a study published in 1940, of 14,411 autopsies, the incidence of renal infarction was 1.4% [2]. In a later study in 2006, of 250,000 patients, only 17 (0.007%) were diagnosed with acute renal infarction [3].

## Case presentation

**Figure 1:**
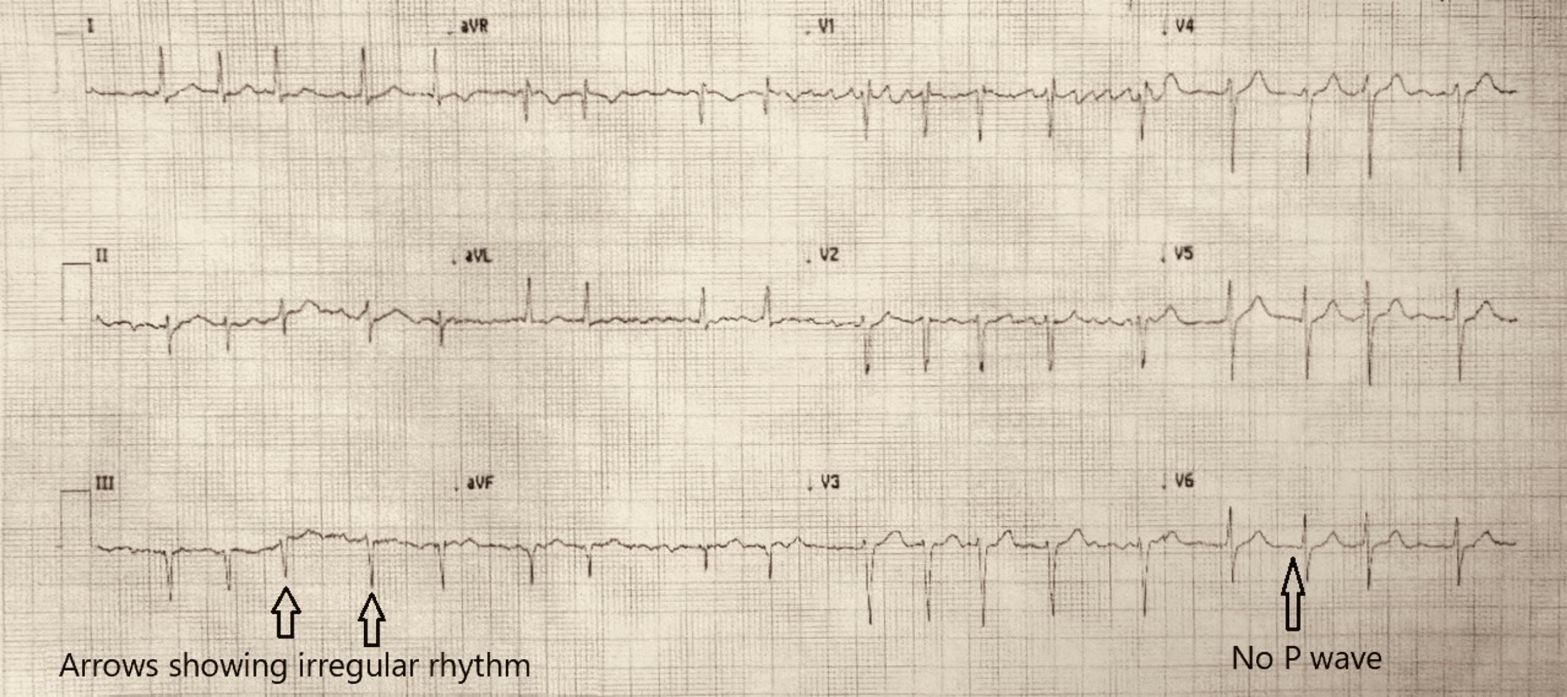
Electrocardiogram showing a new diagnosis of atrial fibrillation. Arrows located in lead III indicate an irregularly irregular rhythm as indicated by R-to-R interval variation. The arrow in lead V6 elucidates the lack of P-waves preceding the QRS intervals.

A 68-year-old woman, with no contributing past medical history, was referred to the emergency department by her general practitioner (GP) due to left flank pain. The patient noted that her pain began approximately two weeks ago; however, it continued to intensify and worsened. She localized the pain to her left flank and noted that it radiated to her left lower quadrant. She described the pain as a dull and constant pain, without any exacerbating or alleviating factors. She quantified the pain as a 4/10 in intensity. The patient noted that she had been experiencing normal bowel movement patterns and did not have any reported urinary symptoms. On physical examination, it was noted that her pulse was irregularly irregular, while auscultation of her heart revealed normal S1/S2 sounds. Her lungs were clear with no added sounds, and the abdomen and flanks were soft and non-tender to palpation. Her blood pressure, respiratory rate, oxygenation, and temperature observations were normal. An electrocardiogram (ECG) revealed a new diagnosis of atrial fibrillation (Figure 1).

Her blood tests were unremarkable, including normal WCC, CRP, renal function, and urine dip (Tables 1, 2).

**Table 1:** Blood tests showing normal WCC, CRP, urea, and creatinine. WCC: white cell count.

Test	Result	Normal range
WCC	10.3 × 10^9^/L	4.0-11.0 × 10^9^/L
CRP	3.1 mg/l	0-5.0 mg/l
Urea	3.9 mmol/l	2.5-7.8 mmol/l
Creatinine	81 umol/l	40-90 umol/l

**Table 2:** Urine dip showing no signs of infection.

Urine dip	Results
Leukocyte	Negative
Nitrate	Negative
Blood	Negative
Color	Light yellow

**Figure 2:**
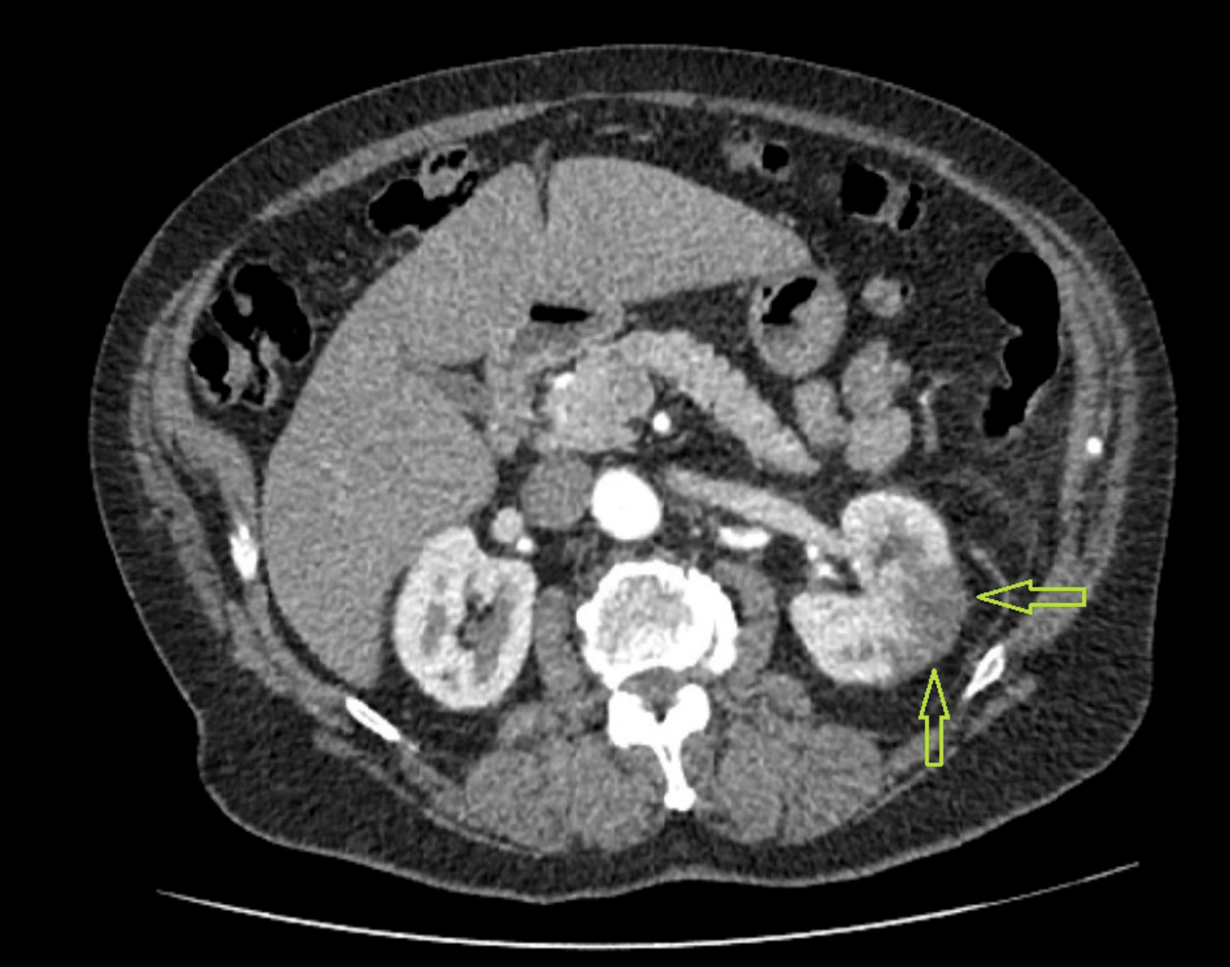
CT angiography of the abdomen and pelvis with IV contrast (contrast-enhanced CT) showing left renal infarct. Arrows showing a wedge-shaped area of decreased attenuation in the left kidney, which is indicative of a renal infarct.

A CT angiography of the abdomen and pelvis with IV contrast (contrast-enhanced CT) was requested for the patient, which showed left renal Infarction (Figure 2).

A vascular consultation was made, and they advised on commencing the patient on direct oral anti-coagulants and co-codamol for pain relief. The patient was discharged with an outpatient clinic review by the vascular team.

## Discussion

This case illustrates how new atrial fibrillation can cause renal thromboembolic events, including renal infarction. Patients typically present with acute flank pain accompanied by abdominal or back pain [4]. Sometimes, the pain is accompanied by hematuria, nausea, and fever, mimicking other more common renal or gastrointestinal disorders such as nephrolithiasis or gastroenteritis. Although these features are common in renal infarction, they are non-specific and not diagnostic. A contrast-enhanced CT abdomen is widely available and can establish a diagnosis of renal infarction in suspected patients [5].

A previously published case report illustrated renal infarction in a patient with normal sinus rhythm on initial ECG, later found to have paroxysmal atrial fibrillation. Thus, in the absence of previous cardiac issues, atrial fibrillation should be investigated with heart rhythm monitoring to look for a potential cardiac source of embolus [6]. 

In the presence of renal infarction, it is crucial to reinstate renal blood flow to prevent potential renal failure. The treatment of renal infarction is based on anti-coagulants, and the duration of treatment depends on the etiology [7].

In addition, this case report details the concern for vascular renal pathologies, including acute renal infarction in newly diagnosed atrial fibrillation presenting with flank pain of unknown etiology; hence, a clear understanding of the patient's complaints and performing relevant investigations to confirm the diagnosis will eventually lead to optimal patient outcomes.

## Conclusions

This case underscores the importance of considering renal infarction as a potential diagnosis in patients who present with unexplained flank pain, especially if they also have new-onset atrial fibrillation. Although renal infarction is rare, if it is not diagnosed and treated promptly, it can lead to significant complications such as chronic kidney disease and renovascular hypertension. Early recognition through appropriate imaging and anticoagulation therapy is crucial to prevent long-term complications. This case emphasizes the importance for clinicians to remain vigilant about the possibility of renal infarction in patients with thromboembolic risk factors, ensuring timely diagnosis and treatment for the best patient outcomes.
